# Nanomolar phosphate supply and its recycling drive net community production in the subtropical North Pacific

**DOI:** 10.1038/s41467-021-23837-y

**Published:** 2021-06-08

**Authors:** Fuminori Hashihama, Ichiro Yasuda, Aki Kumabe, Mitsuhide Sato, Hiroshi Sasaoka, Yosuke Iida, Takuhei Shiozaki, Hiroaki Saito, Jota Kanda, Ken Furuya, Philip W. Boyd, Masao Ishii

**Affiliations:** 1 Department of Ocean Sciences, Tokyo University of Marine Science and Technology, Tokyo, Japan; 2 Institute for Marine and Antarctic Studies, University of Tasmania, Hobart, Australia; 3 Atmosphere and Ocean Research Institute, The University of Tokyo, Chiba, Japan; 4 Department of Aquatic Bioscience, Graduate School of Agricultural and Life Sciences, The University of Tokyo, Tokyo, Japan; 5 Graduate School of Fisheries and Environmental Sciences, Nagasaki University, Nagasaki, Japan; 6 Atmosphere and Ocean Department, Japan Meteorological Agency, Tokyo, Japan; 7 Graduate School of Science and Engineering, Soka University, Tokyo, Japan; 8 Meteorological Research Institute, Japan Meteorological Agency, Ibaraki, Japan

**Keywords:** Element cycles, Marine chemistry

## Abstract

Seasonal drawdown of dissolved inorganic carbon (DIC) in the subtropical upper ocean makes a significant contribution to net community production (NCP) globally. Although NCP requires macronutrient supply, surface macronutrients are chronically depleted, and their supply has been unable to balance the NCP demand. Here, we report nanomolar increases in surface nitrate plus nitrite (N+N, ~20 nM) and phosphate (PO_4_, ~15 nM) from summer to winter in the western subtropical North Pacific. Molar ratios of upward fluxes of DIC:N+N:PO_4_ to the euphotic zone (< 100 m) were in near-stoichiometric balance with microbial C:N:P ratios (107~243:16~35:1). Comparison of these upward influxes with other atmospheric and marine sources demonstrated that total supply is largely driven by the other sources for C and N (93~96%), but not for P (10%), suggesting that nanomolar upward supply of P and its preferential recycling play a vital role in sustaining the NCP.

## Abstract (web summary)

Primary productivity in the oligotrophic ocean sustains Earth’s ecosystems, but nutrient concentrations are vanishingly low. Here the authors measure nanomolar macronutrient concentrations in the North Pacific and find that net community production is sustained through high rates of phosphorus recycling.

## Introduction

In the pelagic realm of the subtropical ocean, macronutrient concentrations are chronically low^1–3^ and frequently below the detection limits of conventional analyses for nitrate plus nitrite (N+N, 50 nM) and phosphate (PO_4_, 30 nM)^4^. Despite such severe oligotrophic regimes, net community production (NCP) is modestly autotrophic^5^, and annual NCP in the euphotic zone of the subtropical regions (15~30°N/S), which was estimated by a variety of methods, ranged from 1.0 to 3.3 mol C m^−2^ y^−1^^6^. Although the NCP requires corresponding amounts of macronutrient supply from below the euphotic zone, no evidence has been found for the substantial supply of macronutrients into the euphotic zone^7,8^. Based on a calculation using microbial C:N (6~11) and C:P ratios (107~243) of *Prochlorococcus*, *Synechococcus*, and pico- and nano-sized eukaryotes collected from subtropical waters^9,10^, macronutrient supply of 9.1 × 10^−2^~5.5 × 10^−1^ mol N m^−2^ y^−1^ and 4.1 × 10^−3^~3.1 × 10^−2^ mol P m^−2^ y^−1^ is required to support the estimated range of NCP (1.0~3.3 mol C m^−2^ y^−1^)^6^. In the central subtropical North Pacific, the seasonal drawdown of the integrated nitrate stocks in the upper 250 m was stoichiometrically reconciled with the annual NCP^8^. However, there are issues with the calculation since nitrate drawdown occurred below the euphotic zone (> 100 m), whereas the majority of NCP occurred in the surface mixed layer shallower than 100-m depth.

Here, we revisit the previously reported imbalance between macronutrient supply and NCP demands in the euphotic zone, using data of sensitive technique-derived nanomolar macronutrient concentrations and their vertical fluxes collected from the western subtropical North Pacific. Given that marine N_2_ fixation and atmospheric N and P depositions represent additional N and P sources to the euphotic zone^11–13^, we summarize the annual budgets of N and P along with C (vertical DIC influx plus atmospheric CO_2_ influx) to quantify C, N, and P stoichiometric requirements need to support annual NCP. Our budgetary analyses conclude that annual P supply is stoichiometrically deficient compared to those of C and N and thus the annual NCP must be ultimately sustained by preferential recycling of P through microbially mediated metabolic processes^14–19^.

## Results and discussion

### Seasonal drawdown of inorganic C, N, and P

**Fig. 1:**
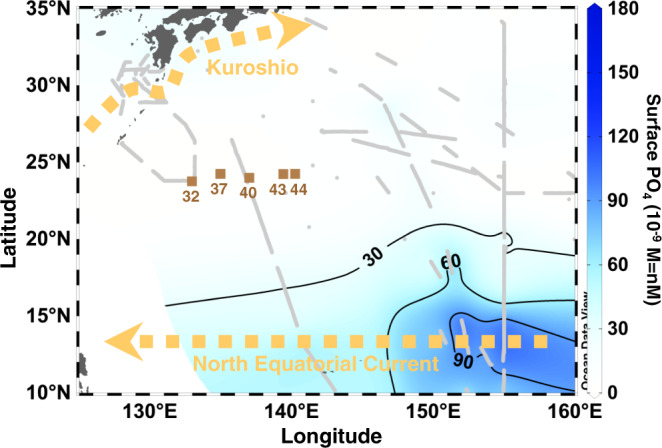
Study areas and sampling stations. Stations are indicated by the brown squares. Typical flow paths of Kuroshio and North Equatorial Current are depicted by the yellow dashed arrows. The background contour shows surface (< 10 m) nanomolar phosphate (PO_4_) concentrations reported in our previous studies^20,21^. The gray dots and gray lines denote sampling stations and underway continuous sampling tracks for the nanomolar PO_4_, respectively.

**Fig. 2:**
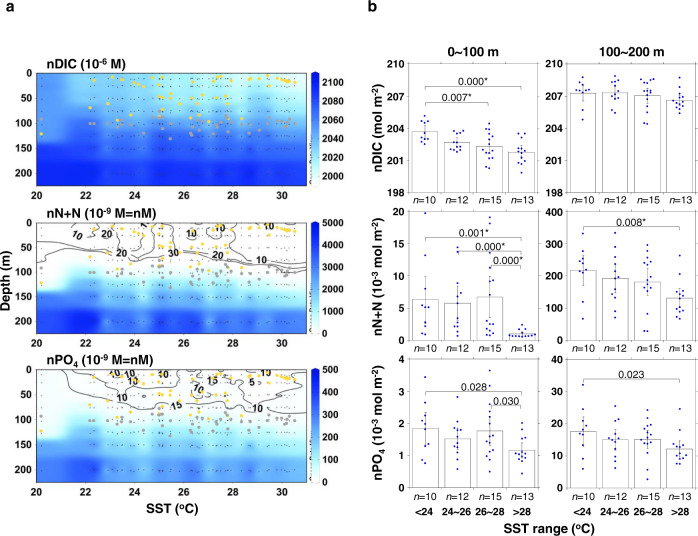
Distribution of the salinity (39.41) normalized dissolved inorganic carbon (nDIC), nitrate plus nitrite (nN+N), and phosphate (nPO_4_) over sea surface temperature (SST) in the upper 200 m of the western subtropical North Pacific. **a** Vertical distributions of nDIC, nN+N, and nPO_4_ concentrations plotted against SST. The small black dots denote the sampling depths. The yellow and gray solid circles indicate the mixed layer depth (MLD) and euphotic zone depth (EZD), respectively. The black contour lines in the nN+N and nPO_4_ panels represent nanomolar gradients of ~30 nM (10 nM interval) and ~15 nM (5 nM interval), respectively. **b** Integrated stocks of nDIC, nN+N, and nPO_4_ in four different SST regimes within the 0~100 m (left) and 100~200 m (right) layers. Bar charts represent mean values of the integrated stocks indicated as blue dots. The error bars denote the 95% confidence interval (CI). Significant differences (two-sided Kruskal-Wallis test, *P* < 0.05) in the mean stocks between the SST regimes are depicted just above the bars. Significant differences of multiple comparisons with the Dunn’s procedure after the Kruskal-Wallis test (*P* < 0.0083) are marked with an asterisk (*).

We conducted shipboard time-series measurements of physical and biogeochemical variables at five stations along a 24°N zonal transect (133.0~140.3°E) between April 2014 and May 2019 (Fig. 1 and Supplementary Table 1). These stations are > 500 km away from the Japanese archipelago, Kuroshio, and North Equatorial Current, and characterized by severe depletion of surface PO_4_ (< 30 nM) along with N+N (< 50 nM)^20,21^. Potential temperature-salinity (T-S) diagram at these stations over the study period showed no distinct east-west trend in physical properties of water masses (Supplementary Fig. 1) as potential temperature and salinity at 10-, 100-, and 200-m depths were not significantly different among the stations (*P* > 0.05). The concentrations of DIC, N+N, and PO_4_ were each normalized using a mean salinity (34.91) to take into account the effects of evaporation and precipitation on dissolved constituents^22^ in the upper 200 m of the study region (Fig. 2a). These normalized concentrations (nDIC, nN+N, and nPO_4_) were plotted against sea surface temperature (SST) at 10-m depth, which showed a clear seasonal trend from 20.21 °C in January to 30.51 °C in July, covering the range of the monthly mean SST derived from satellite observation during the study period (Supplementary Fig. 2a, b). The nDIC, nN+N, and nPO_4_ generally increased with depth but exhibited trends of decreasing concentrations from the low-SST regime to the high-SST regime (Fig. 2a). Interestingly, nN+N and nPO_4_ in the upper 100 m exhibited variability in the nanomolar range (~30 nM) with lower concentrations in the stratified period characterized by the high SST (> 28 °C) and shallow surface mixed layer depth (MLD, < 50 m).

In the surface layer above 100-m depth which corresponds to the winter MLD and mean euphotic zone depth (EZD) (Fig. 2a and Supplementary Fig. 2c, d), inventories of nDIC, nN+N, and nPO_4_ were significantly lower in the warmer (SST > 28 °C) regime than the cooler (SST < 24 °C) regime (*P* < 0.05) (Fig. 2b). The differences between the 0~100 m inventories of the cooler and warmer regimes (i.e., winter-summer differences) were 1.9, 5.3 × 10^−3^, and 6.8 × 10^−4^ mol m^−2^ for nDIC, nN+N, and nPO_4_, respectively. The seasonal drawdown of nDIC from winter to summer (1.9 ± 0.8 mol m^−2^), which typically contributes a substantial proportion of annual NCP^23,24^, was within the bounds of previous estimates of annual NCP in the subtropical oceans (1.0~3.3 mol C m^−2^ y^−1^)^6^. In the layer between 100 and 200 m, the inventories of nDIC were not significantly different between any pair of SST regimes (*P* > 0.05), while those of nN+N and nPO_4_ were significantly lower in the regime of SST > 28 °C than that of SST < 24 °C (*P* < 0.05), suggesting that seasonal drawdown of macronutrients in the 100~200 m layer was not linked to the NCP.

### Upward fluxes of inorganic C, N, and P

**Fig. 3:**
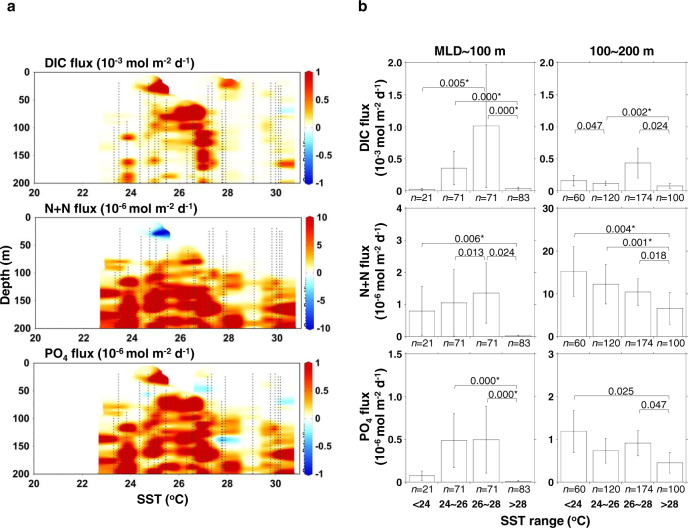
Vertical fluxes of dissolved inorganic carbon (DIC), nitrate plus nitrite (N+N), and phosphate (PO_4_) over sea surface temperature (SST) in the layer between the mixed layer depth (MLD) and 200-m depth of the western subtropical North Pacific. **a** Vertical profiles of DIC, N+N, and PO_4_ fluxes plotted against SST. The small black dots denote the depths where *K*_*z*_ data were obtained. **b** Mean fluxes of DIC, N+N, and PO_4_ in four different SST regimes within the MLD~100 m (left) and 100~200 m (right) layers shown in **a**. The error bars denote the 95% confidence interval (CI). Significant differences (two-sided Kruskal-Wallis test, *P* < 0.05) in the mean fluxes between the SST regimes are stated just above the bars. Significant differences of multiple comparisons with the Dunn’s procedure after the Kruskal-Wallis test (*P* < 0.0083) are marked with an asterisk (*).

We estimated vertical fluxes of DIC, N+N, and PO_4_ in the upper 200 m of the study region between June 2016 and May 2019 (Fig. 3a and Supplementary Table 1), using the vertical concentration gradients of DIC, N+N, and PO_4_ and the vertical diffusivity (*K*_*z*_) determined using fast-response thermistors (“Methods”). Here, we regarded the mean fluxes in the MLD~100 m and 100~200 m layers as upward fluxes into the mixed layer and euphotic zone, respectively (Fig. 3b), based on the theory proposed by previous macronutrient flux studies^25–27^. The DIC, N+N, and PO_4_ upward fluxes generally showed positive values even in the MLD~100 m layer, and were higher in SST regimes of < 28 °C than the > 28 °C regime, particularly for N+N and PO_4_ fluxes. The positive upward fluxes of N+N and PO_4_ in the MLD~100 m layer were ascribed to the nanomolar concentration gradients of N+N and PO_4_ (Supplementary Fig. 3). Historically, it had been reported that there is no upward nitrate supply to the mixed layer of the subtropical North Pacific due to the lack of a vertical concentration gradient in nitrate in the euphotic zone^28,29^. However, our observations revealed that N+N and PO_4_ were supplied to the mixed layer particularly in the cool period with SST < 28 °C, and thus potentially support the NCP.

We present several additional lines of evidence for seasonal linkages between upward macronutrient supply and microbial dynamics. We found that the increases in upward macronutrient fluxes into the euphotic zone from summer (SST > 28 °C) to winter (SST < 24 °C) (Fig. 3) likely explain the corresponding increases in inventories of total particulate P (TPP) in the 0~100 m layer from summer to winter (Supplementary Fig. 4a, b). Since TPP is a useful metric for living microbial biomass^3,9^, these seasonal trends suggest that the enhanced upward macronutrient supply induced an increase in microbial biomass. In addition, the chemical composition of TPP showed that, although nucleic acid P was a major component of TPP over the study period, the proportion of orthophosphate (PO_4_) in TPP was significantly higher in the cool period (24 °C < SST < 26 °C) than in the warm period (SST > 28 °C) (Supplementary Fig. 4c, d); this finding is evidence for constant microbial uptake (or adsorption onto the cell surface^30^ and accumulation in the periplasm^31^) of PO_4_ that was supplied upward from the subsurface layer below 100 m over the cool period. We also found a seasonal succession of major microbial groups in the upper 100 m of the study region from pico- and nano-sized eukaryotes in winter to cyanobacteria (*Prochlorococcus* and *Synechococcus*) in summer and vice versa (Supplementary Fig. 5). Since the microbial C biomass is largely contributed by cyanobacteria in low-macronutrient oligotrophic regimes^9,10^, the higher proportion of C biomass of cyanobacteria relative to eukaryotes in summer than winter was likely associated with the lower upward fluxes of macronutrients in summer than winter (Fig. 3).

### Magnitude of NCP is driven by rapid P recycling

**Fig. 4:**
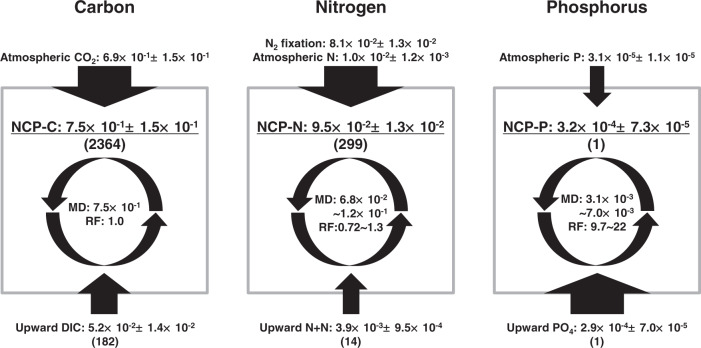
The C, N, and P requirements for annual net community production (NCP). Each box represents the water column (0~100 m depth). The upward and downward black arrows with black text indicate the influxes (mol m^−2^ y^−1^) from the subsurface layer (100~200-m depth) and atmosphere (including marine N_2_ fixation), respectively. The width of the arrows indicates the relative magnitudes of influxes to the total influx for each element. The C, N, and P requirements for annual NCP (mol m^−2^ y^−1^) are denoted using underlined text. Stoichiometric ratios of the NCP-C:NCP-N:NCP-P and upward dissolved inorganic carbon (DIC):nitrate plus nitrite (N+N):phosphate (PO_4_) are denoted in parentheses. The microbial demands based on the C:N:P ratio of subtropical microbes^9,10^ (MD, mol m^−2^ y^−1^) and the recycling factors (RF = MD/NCP, no unit) are represented within the circle of arrows. The errors of the stated values denote the 95% confidence interval (CI). The procedures for calculating the stated values are described in the main text and “Methods”.

The annual NCP in the subtropical ocean is balanced by annual downward fluxes of particulate and dissolved organic C including those through zooplankton migration, indicating that net C uptake and downward export are at a steady state over the annual cycle^6,32^. Assuming the steady state at the annual scale, we estimated C, N, and P requirements for the annual NCP (NCP-C, -N, and -P) in the 0~100 m layer (Fig. 4), using total annual influxes of C, N, and P that include upward fluxes of DIC, N+N, and PO_4_ from the subsurface layer (100~200 m) based on in situ observation (Fig. 3b), atmospheric CO_2_ flux and N and P depositions, and marine N_2_ fixation (Supplementary Fig. 6 and “Methods”). The NCP-C, -N, and -P were 7.5 × 10^−1^, 9.5 × 10^−2^, and 3.2 × 10^−4^ mol m^−2^ y^−1^, respectively. The NCP-C (7.5 × 10^−1^ ± 1.5 × 10^−1^ mol m^−2^ y^−1^) is comparable to the lowest estimates of previous studies in the subtropical oceans (1.0~3.3 mol C m^−2^ y^−1^)^6^. This low estimate may be due to the noninclusion of upward supply of DIC during the cool period with SST < 23 °C (Fig. 3) and/or the passage of typhoon and eddies^33^ when shipboard observation could not be performed. The missing DIC supply is also inferred from the lower value of NCP-C (7.5 × 10^−1^ ± 1.5 × 10^−1^ mol m^−2^ y^−1^) than the seasonal drawdown of nDIC from winter to summer (1.9 ± 0.8 mol m^−2^, Fig. 2b).

Interestingly, the stoichiometric ratio of the annual NCP-C, -N, and -P (2364:299:1) (Fig. 4) was higher than the C:N:P ratio of subtropical microbes (107~243:16~35:1)^9,10^, indicating that the P influx was (stoichiometrically) lower than the C and N influxes. Regarding the annual upward flux, the ratios of DIC:N+N:PO_4_ (182:14:1) were in near-stoichiometric balance to the C:N:P ratio of subtropical microbes compared to that of annual NCP. In addition, there were substantial annual influxes of C and N from the atmosphere and marine N_2_ fixation. The contribution of marine N_2_ fixation reached 85% in the NCP-N. This is in line with recent findings at the Hawaii Ocean Time-Series (HOT) in the subtropical North Pacific that the annual rate of marine N_2_ fixation is stoichiometrically reconciled with the annual NCP^34^. Meanwhile, the annual P influx from the atmosphere was almost negligible and the upward marine P influx accounted for 90% of the total P influx. When only considering inorganic C-N-P forms, the total influx of P thus did not meet microbial P demand.

As an alternative to PO_4_, dissolved organic phosphorus (DOP) is an important P source for the microbes in the western subtropical North Pacific^3,35^. However, a significant seasonal change in the salinity (34.91) normalized DOP inventory (nDOP) was not observed in the 0~100 m layer from the cooler (SST < 24 °C) regime to warmer (SST > 28 °C) regime (Supplementary Fig. 7a, b). Furthermore, the vertical DOP flux was smaller than the vertical PO_4_ flux due to its small concentration gradient, and the annual vertical DOP flux was not significantly different from zero in both the MLD~100 m (4.8 × 10^−6^ ± 2.6 × 10^−5^ mol m^−2^ y^−1^) and 100~200 m (−2.4 × 10^−5^ ± 3.0 × 10^−5^ mol m^−2^ y^−1^) layers (Supplementary Figs. 3 and 7c, d). Several studies reported the importance of lateral DOP supply into the subtropical gyres from the gyre margins^36,37^. However, the lateral supply of DOP along with PO_4_ into the study area was negligible, because the compiled data on our previous studies^3,38^ and this study demonstrated that inventories of total dissolved P (TDP = DOP + PO_4_) in the 0~100 m layer of the 24°N zonal transect were not significantly different from those of 20~24°N and 24~30°N in the western subtropical North Pacific (*P* > 0.05, Supplementary Fig. 8). Since the DOP supply and its microbial demand are spatiotemporally balanced, nanomolar vertical PO_4_ supply to the euphotic zone and rapid internal recycling of P, relative to C and N, within the euphotic zone are suggested to be major processes driving the NCP. This perspective is also supported by a range of biogeochemical studies which showed that organic P is preferentially remineralized compared to organic N and C (i.e., the shorter turnover time of organic P than organic N and C) in the various subtropical regions of the global ocean^14–19^.

Our datasets on nanomolar concentrations of N+N and PO_4_ make their upward supply to not only the euphotic zone but also the mixed layer conspicuous; however, it is currently problematic to measure the recycling rates (i.e., uptake or regeneration rates at a steady state) of N and P using the conventional stable or radioisotopic methods^39,40^ which have some issues with bottle effects, filtration effects, and incubation time as well known in the primary production estimates using ^13^C and ^14^C^41^. Hence in our study, we must constrain to assess the recycling of N and P by comparing microbial demand (MD) with NCP-N and -P. Assuming that the C:N (6~11) and C:P (107~243) ratios of subtropical microbes^9,10^ are representatives of MD ratios, the annual MD-N and -P are estimated to be 6.8 × 10^−2^~1.2 × 10^−1^ mol N m^−2^ y^−1^ and 3.1 × 10^−3^~7.0 × 10^−3^ mol P m^−2^ y^−1^, respectively, based on the NCP-C (7.5 × 10^−1^ mol C m^−2^ y^−1^) (Fig. 4). The MD-N is comparable to the NCP-N quantified in situ (9.5 × 10^−2^ mol N m^−2^ y^−1^), while the MD-P is 9.7~22-fold higher than the NCP-P quantified in situ (3.2 × 10^−4^ mol P m^−2^ y^−1^). In other words, the internal recycling rate of N is similar to that of C, but that of P is 9.7~22-fold faster than that of C (referred to as recycling factors (RF) in Fig. 4).

In this study, we found that nanomolar macronutrient supply contributes to the NCP in the mixed layer of the western subtropical North Pacific. Our C-N-P budgetary analysis showed that the NCP in the euphotic zone is driven by the stoichiometrically P-deficient C-N-P influx. As a range of biogeochemical studies suggested preferential remineralization of organic P relative to organic C and N^14–19^, the rapid internal recycling of P in the euphotic zone could play a vital role in sustaining the NCP in the oligotrophic subtropical ocean. Since marine N_2_ fixation and atmospheric N deposition are projected to increase with ocean warming^42^ and anthropogenic activities^12,43^, respectively, the P supply and recycling would become more influential in controlling the NCP in the subtropical ocean in the coming decades.

## Methods

### Observations and sampling

Time-series observations in the western subtropical North Pacific were conducted aboard the R/V Keifu-maru and R/V Ryofu-maru of Japan Meteorological Agency (JMA). We occupied five stations along a 24°N zonal transect from 133.0°E to 140.3°E during 13 cruises between April 2014 and May 2019 (Fig. 1 and Supplementary Table 1). Every cruise generally occupied Stations 32, 37, 40, and 44, but the observations were not conducted at Station 44 of the KS15-01cruise and at Stations 37 and 44 of the KS16-01cruise. During the KS16-01cruise, Station 43 was occupied instead of Station 44.

At all stations, water samples were collected from 9 depths (0, 10, 25, 50, 75, 100, 125, 150, and 200 m) in the upper 200 m of the water column using an acid-cleaned bucket (for surface layer) and 12-L acid-cleaned Niskin bottles (General Oceanics) mounted on a conductivity-temperature-depth (CTD) system (Sea-Bird Electronics). MLD was determined using the temperature profile obtained by the CTD sensor, as the depth where the potential temperature was 0.2 °C less than that at 10-m depth^44^. Vertical profiles of photosynthetically active radiation (PAR) were measured using a DEFI2-L PAR sensor (JFE Advantech) to determine EZD, defied as the depth of 1% PAR relative to surface PAR^45^. The PAR observations were conducted at most stations, but when the station was occupied during nighttime, PAR was measured in the daytime at a nearby site (within 132 km). To confirm the seasonal trend of SST in the study region, we used data on monthly mean satellite SST (MODIS Aqua Level 3 SST MID-IR Monthly 4 km Nighttime V2019.0)^46^ within the area between 23.5~24.5°N and 132.5~140.5°E for the period from April 2014 to May 2019.

### Determination of dissolved constituents

Water samples for DIC, N+N, PO_4_, and DOP were taken throughout the study period (Supplementary Table 1). Subsamples for DIC analyses were collected into 250-mL borosilicate glass bottles with ground glass stoppers lubricated with Apiezon L grease. They were poisoned with mercury (II) chloride and analyzed by a CO_2_ extraction-coulometry method using a DIC analyzer manufactured by Nippon ANS. To establish the identical concentration scale across the cruises, we used numbers of batches of Certified Reference Material for DIC and total alkalinity analyses provided by A.G. Dickson at the University of California, San Diego^47^. The precision of DIC analysis was 2.0 × 10^−6^ ± 3.4 × 10^−6^ M (*n* = 194).

Water samples for N+N and PO_4_ were collected in 30-mL polypropylene tubes for nanomolar (10^−9^ M = nM) level analysis and in 10-mL polymethylpentene tubes for micromolar (10^−6^ M) level analysis. The samples for the nanomolar level analysis were frozen and stored at −20 °C until the analyses were conducted onshore, and those for micromolar level analysis were immediately processed onboard. The nanomolar levels of N+N and PO_4_ (< 1000 nM) were determined using an automated liquid waveguide spectrophotometric system equipped with 50- and 100-cm liquid waveguide capillary cells (LWCC, World Precision Instruments), respectively^2^. The detection limits for nitrate, nitrite, and PO_4_ were 3, 2, and 3 nM, respectively^48^. The reproducibility of nitrate, nitrite, and PO_4_ at 100 nM had a coefficient of variation (CV) of 2% (*n* = 10)^48^. In the calculation of vertical fluxes, water column integrated stocks, stoichiometric molar ratio, and DOP concentration (see below), the N+N and PO_4_ concentrations below the detection limits (11% and 9% of all data, respectively) were regarded as 5 and 3 nM, respectively. The micromolar concentrations (> 1000 nM) of N+N were determined using a conventional autoanalyzer (SEAL Analytical).

DOP water samples were collected in 30-mL polypropylene tubes after removing particulate matter by filtering through precombusted Whatman GF/F filters. The samples were frozen and stored at −20 °C until the analyses were performed onshore. DOP concentrations were determined by a sensitive method^35^. TDP was quantified by liquid waveguide spectrophotometry for PO_4_^2^ after the filtrate sample was oxidized with acid persulfate^49^. The oxidation efficiency of 100 nM organic P analogs (D-Glucose-6-phosphate sodium salt, Sodium tripolyphosphate pentabasic, and Sodium phosphonoformate tribasic hexahydrate from Sigma-Aldrich) was 98~104% and the CV of the measurements was 1% (*n* = 9). DOP concentrations were derived from the difference between TDP and PO_4_ concentrations.

### Estimating vertical fluxes of dissolved constituents

We estimated the vertical fluxes of DIC, N+N, PO_4_, and DOP in the layer between the MLD and 200-m depth during the six cruises between June 2016 and May 2019, since data on vertical diffusivity (*K*_*z*_) were available for this period (Supplementary Table 1). We excluded the upward and downward fluxes of DIC, N+N, and PO_4_ within the mixed layer from our analyses because these are not influxes from outside the mixed layer and do not support the NCP in the mixed layer at a steady state. To calculate the vertical fluxes, we used the following equation:

1 $${F}_{X}={K}_{z}(\partial X/\partial z)$$

where *F* is the vertical flux, *X* is the dissolved constituents (DIC, N+N, PO_4_, or DOP), and *∂X*/*∂z* (mol m^−4^) is the vertical gradient of the dissolved constituents. The *K*_*z*_ (cm^2^ s^−1^) was estimated using CTD-attached fast-response thermistors (AFPO7; Rockland Scientific International Company)^50–53^, and was calculated using the following equation:

2 $${K}_{z}=0.2\varepsilon {N}^{-2}$$

where *ε* (W kg^−1^) is turbulent kinetic energy dissipation rate and *N* (s^−1^) is buoyancy frequency. As *X* flux was obtained from the 5-m interval *K*_*z*_ data, we also used the 5-m interval *∂X*/*∂z* data which was derived from the linearly interpolated concentrations of *X* between sampling depths (Supplementary Fig. 3).

### Determination of particulate P and its chemical composition

Water samples for TPP were taken throughout the study period (Supplementary Table 1). Sample processing and analysis were followed by the procedure for the sensitive determination of TPP^54^. Water volumes of 200 mL were filtered through precombusted acid-washed GF/F filters. The samples were stored at −20 °C until onshore measurement. TPP measurements were performed by liquid waveguide spectrophotometry for PO_4_^2^ after a wet oxidation treatment using 3% potassium persulfate (Wako)^55^. The sensitive TPP measurements using ultraoligotrophic water samples had a CV of 4.3% (*n* = 5)^54^.

Water samples for TPP composition were collected from three depths (10, 50, and 100 m) at the stations during the five cruises between June 2016 and December 2017 (Supplementary Table 1). The TPP composition was determined by a combination method of chemical fractionation^56^ and liquid waveguide spectrophotometry^2,54^. Water volumes of 2.3 L were filtered through precombusted acid-washed GF/F filters. The samples were stored at −20 °C until onshore measurement. According to the chemical fractionation procedures involving trichloroacetic acid extraction, hot dilute acid treatment, charcoal treatment, and organic solvent extraction, TPP was divided into orthophosphate, sugar P, nucleotide P, nucleic acid P, lipid P, acid-soluble poly P, acid-insoluble poly P, and residual P^56^. After converting the P in each chemical fraction to PO_4_ with a wet oxidation treatment^54,55^, the P amount was determined by liquid waveguide spectrophotometry for PO_4_^2^. In the liquid waveguide spectrophotometric analysis, the treated individual solutions with the GF/F filter (no sample on it) were used as blank and standard matrices. The CV of the mean P concentration in each fraction (*n* = 3) accounted for < 10% of the TPP concentration, indicating that the proportion of each P fraction in TPP was reliable.

### Flow cytometry for microbial analysis

Water samples for flow cytometry (FCM) were taken from the seven cruises between April 2014 and June 2016 (Supplementary Table 1). The 5-mL samples were fixed with 1% (v/v) glutaraldehyde. The fixed samples were frozen in liquid nitrogen and then stored at −80 °C until onshore analysis. We analyzed the samples using a flow cytometer (CyFlow Space, Partec)^57^. *Prochlorococcus*, *Synechococcus*, and pico- and nano-sized eukaryotes were identified using the conventional protocols^58^. Gating strategy using forward light scatter, side light scatter, orange fluorescence, and red fluorescence was described in our previous studies^57,59^. The gating was processed with FloMax ver. 2.3 (Partec). The C biomass of these microbial groups was estimated by multiplying the FCM-derived cell abundance by the published cellular C quota. We used the cellular C quota of 5.2 × 10^−15^, 3.0 × 10^−14^, and 3.9 × 10^−13^ mol C cell^−1^ for *Prochlorococcus*, *Synechococcus*, and eukaryotes, respectively^10^. The contributions of the C biomass of individual groups to the total C biomass of these groups were compared (Supplementary Fig. 5b).

### Estimating C, N, and P requirements need to support annual NCP

The C, N, and P requirements for annual NCP in the 0~100 m layer of the study region were estimated assuming a steady state over the annual scale by the following equation:

3 $${\rm{NCP}}{\hbox{-}}{\rm{Y}}={{\rm{Fup}}}_{\rm{Y}}+{{\rm{Fat}}}_{\rm{Y}}$$

where Y is C, N or P. Fup_C_, Fup_N_, and Fup_P_ are annual upward influxes of DIC, N+N, and PO_4_, respectively. Fat_C_, Fat_N_, and Fat_P_ are annual influxes of atmospheric CO_2_, the sum of marine N_2_ fixation and atmospheric N deposition, and atmospheric P deposition, respectively. In the central part (20~30°N) of the western subtropical North Pacific, lateral fluxes of C, N, and P are generally negligible, because no significant horizontal concentration gradients of DIC, N+N, and PO_4_ in the surface layer were observed previously^3,20,21,23^ (that of TDP was described in the main text with Supplementary Fig. 8). Nutrient transport by vertically migrating diatoms is another important source sustaining the NCP^60,61^. However, our microscopic observation using the Utermöhl method^62^ revealed that abundance of the migrating diatoms (*Rhizosolenia* spp.) was consistently low (1.0 ± 0.3 cells L^−1^, *n* = 234) in the upper 200 m of the study area during the seven cruises between April 2014 and June 2016 (Supplementary Table 1) and thus we did not include the macronutrient flux by the migrating diatoms. In the calculation of annual fluxes, the numbers of months in four different SST regimes were first determined using the monthly mean satellite SST (Supplementary Fig. 2b), and then the annual fluxes were estimated by summarizing the integrated fluxes of four different SST regimes (Supplementary Fig. 6).

The integrated upward influx of DIC, N+N, or PO_4_ in each SST regime to estimate Fup_C_, Fup_N_, and Fup_P_ was based on in situ data shown in Fig. 3b, and it was calculated by multiplying mean daily upward flux in the subsurface layer (100~200 m, Fig. 3b) by the numbers of days in each SST regime.

The integrated air-sea CO_2_ flux in each SST regime was calculated by multiplying the mean monthly air-sea CO_2_ flux by the numbers of months in each SST regime. The monthly air-sea CO_2_ fluxes from April 2014 to May 2019 within the area between 23.5~24.5°N and 132.5~140.5°E were estimated using the *p*CO_2_ diagnostic model^63^. The ocean of the study region acted as CO_2_ sink through most periods except for the SST > 28 °C regime (Supplementary Fig. 6), and totally Fat_C_ showed a positive value (Fig. 4).

The annual flux of marine N_2_ fixation was estimated by using time-series in situ data obtained at the HOT site in the subtropical North Pacific^34^. Since N_2_ fixation rates derived from the ^15^N_2_ dissolution method tend to be higher than those derived from ^15^N_2_ bubble method^3,34,64^, we used the data from ^15^N_2_ dissolution method in this study. As a large proportion of the N_2_ fixation occurred within the mixed layer^3,65^, we assumed integrated N_2_ fixation rates in the upper 125 m of the HOT site as the integrated N_2_ fixation rates in the 0~100 m layer in this study. The seasonal variation in the N_2_ fixation at the HOT site (7.7 × 10^−5^~3.6 × 10^−4^ mol N m^−2^ d^−1^) is the same order of magnitude as the discretely measured N_2_ fixation with the dissolution method in the euphotic zone (< 103~117 m) of the western subtropical North Pacific (20~23°N and 137°E~160°W) (3.5 × 10^−5^~2.9 × 10^−4^ mol N m^−2^ d^−1^, *n* = 10)^3^. Since the seasonal trend in the N_2_ fixation rates measured from the dissolution method remains unclear at the HOT site and the western subtropical North Pacific, we used a mean N_2_ fixation rate (2.2 × 10^−4^ ± 6.4 × 10^−5^ mol N m^−2^ d^−1^) to calculate the integrated rate in each SST regime.

For atmospheric N and P depositions, in situ data over the oceanic area of the western subtropical North Pacific are limited and their seasonal trends remain unclear. Thus, we used mean deposition rates for bioavailable N (2.7 × 10^−5^ ± 5.7 × 10^−6^ mol N m^−2^ d^−1^) and P (8.4 × 10^−8^ ± 5.4 × 10^−8^ mol P m^−2^ d^−1^) taken from sites along a meridional transect through the western subtropical North Pacific during five cruises^13^ to calculate the integrated rate in each SST regime.

### Calculation and statistical analyses

In this study, water column inventories of all sampled parameters were calculated by the trapezoidal rule. The errors with mean inventory, mean flux, and mean ratio were reported as 95% confidence interval (CI). For the error propagation when calculating mean value with addition, subtraction, or division, we adopted the following three equations, respectively^66^.

4 $$({M}_{1}\pm {E}_{1})+({M}_{2}\pm {E}_{2})=({M}_{1}+{{\rm{M}}}_{2})\pm \sqrt{{E}_{1}^{2}+{E}_{2}^{2}}$$

5 $$({M}_{1}\pm {E}_{1})-({M}_{2}\pm {E}_{2})=({M}_{1}-{M}_{2})\pm \sqrt{{E}_{1}^{2}+{E}_{2}^{2}}$$

6 $$\frac{({M}_{1}\pm {E}_{1})}{({M}_{2}\pm {E}_{2})}=\frac{{M}_{1}}{{M}_{2}}\pm \frac{{M}_{1}}{{M}_{2}}\sqrt{{(\frac{{E}_{1}}{{M}_{2}})}^{2}+{(\frac{{E}_{2}}{{M}_{2}})}^{2}}$$

where *M* is mean and *E* is 95% CI.

Statistical analyses were performed using XLSTAT premium ver. 2018.1.1.61323 (Addinsoft). A Friedman test was used to compare paired (same cruise) values of potential temperature or salinity at each depth between the four stations (except for Station 43). A two-sided Kruskal-Wallis test was used to compare the mean values of biogeochemical parameters between four different SST regimes or three different regional stations. Significance is reported where *P* < 0.05, except for multiple comparisons with the Dunn’s procedure after the Kruskal-Wallis tests. The significance of the multiple comparisons for four different SST regimes is reported where *P* < 0.0083, as the Bonferroni correction was adopted.

### Data visualizations

To draw the distributions and fluxes of biogeochemical variables, we used Ocean Data View (ver. 5.2.1, https://odv.awi.de/), Kaleida Graph (ver. 4.5.1, Synergy Software), and Microsoft PowerPoint for Office 365. The color contours in Figs. 1, 2a, and 3a were drawn by the weighted-average griding of Ocean Data View with *x-y* scale-lengths of 110-110, 65-65, and 40-30, respectively.

### Reporting summary

Further information on research design is available in the Nature Research Reporting Summary linked to this article.

## Supplementary information

Supplementary Information

Reporting summary

## Data Availability

Physical and biogeochemical data used in this study are available in the Supplementary Data 1 File that is deposited in the TUMSAT-OACIS site of Tokyo University of Marine Science and Technology (http://id.nii.ac.jp/1342/00002046/). The data including DIC and micromolar macronutrients that JMA measures routinely are also available from https://www.data.jma.go.jp/gmd/kaiyou/db/vessel_obs/data-report/html/ship/ship_e.php and GLODAPv2 (https://www.glodap.info/). Data on monthly mean satellite SST (MODIS Aqua Level 3 SST MID-IR Monthly 4 km Nighttime V2019.0) were downloaded from the PODAAC site of NASA Earth Data (https://podaac.jpl.nasa.gov/). The monthly data of air-sea CO_2_ fluxes are taken from JMA’s website at https://www.data.jma.go.jp/gmd/kaiyou/english/co2_flux/co2_flux_data_en.html. Source data are provided with this paper.

## Source data

source data
