# Supplementary material for: Nanomolar phosphate supply and its recycling drive net community production in the subtropical North Pacific
Source: Nat Commun. 2021 Jun 8;12:3462. doi: 10.1038/s41467-021-23837-y (PMC8187552; doi:10.1038/s41467-021-23837-y)
Supplement: Supplementary file 1 — Supplementary Information [file 41467_2021_23837_MOESM1_ESM.pdf]

Supplementary information for:

Nanomolar phosphate supply and its recycling drive net community production in the subtropical North Pacific

Hashihama et al.

The file contains:

Supplementary Table 1

Supplementary Figures 1-8

Supplementary Table 1. Ancillary observations

Cruise*	Station	Year	Month	Day	Latitude (°N)	Longitude (°E)	MLD (m)	EZD (m)	SST (°C)	CTD	K_z	DIC	Macro-nutrients	DOP	TPP	TPP composition	FCM phytoplankton
KS14-03	32	2014	4	29	23.76	132.98	28		24.16	×		×	×	×	×		×
KS14-03	37	2014	4	30	24.25	134.98	19	107	23.48	×		×	×	×	×		×
KS14-03	40	2014	4	30	24.00	137.01	14		23.10	×		×	×	×	×		×
KS14-03	44	2014	5	1	24.25	140.25	20	105	22.35	×		×	×	×	×		×
RF14-09	32	2014	11	24	23.75	132.99	91	94	25.01	×		×	×	×	×		×
RF14-09	37	2014	11	24	24.24	134.98	66		25.83	×		×	×	×	×		×
RF14-09	40	2014	11	25	23.99	137.00	14	92	25.12	×		×	×	×	×		×
RF14-09	44	2014	11	26	24.24	140.25	52	81	25.01	×		×	×	×	×		×
KS15-01	32	2015	1	20	23.72	132.99	66	105	22.92	×		×	×	×	×		×
KS15-01	37	2015	1	20	24.23	135.00	125	96	20.21	×		×	×	×	×		×
KS15-01	40	2015	1	21	23.97	137.01	74	105	22.19	×		×	×	×	×		×
RF15-06	32	2015	7	2	23.78	133.00	22	96	30.29	×		×	×	×	×		×
RF15-06	37	2015	7	1	24.25	135.00	31	94	29.47	×		×	×	×	×		×
RF15-06	40	2015	7	1	24.03	137.01	24	105	30.51	×		×	×	×	×		×
RF15-06	44	2015	6	30	24.25	140.29	21	110	30.18	×		×	×	×	×		×
RF15-09	32	2015	11	20	23.73	133.00	49	100	26.76	×		×	×	×	×		×
RF15-09	37	2015	11	20	24.26	135.00	54	107	27.32	×		×	×	×	×		×
RF15-09	40	2015	11	19	24.00	137.00	51	89	27.69	×		×	×	×	×		×
RF15-09	44	2015	11	18	24.26	140.26	95	107	28.35	×		×	×	×	×		×
KS16-01	32	2016	1	17	23.77	132.98	25		23.98	×		×	×	×	×		×
KS16-01	40	2016	1	18	24.00	137.03	94	124	26.28	×		×	×	×	×		×
KS16-01	43	2016	1	19	24.24	139.42	54	115	25.14	×		×	×	×	×		×
KS16-06	32	2016	6	23	23.75	132.99	21	112	30.11	×	×	×	×	×	×	×	×
KS16-06	37	2016	6	22	24.26	135.00	23	102	30.20	×	×	×	×	×	×	×	×
KS16-06	40	2016	6	21	24.00	137.00	16	100	29.79	×	×	×	×	×	×	×	×
KS16-06	44	2016	6	21	24.25	140.25	19	100	29.98	×	×	×	×	×	×	×	×
KS16-09	32	2016	11	21	23.75	132.99	95	121	27.80	×	×	×	×	×	×	×	
KS16-09	37	2016	11	20	24.24	134.99	66	98	26.55	×	×	×	×	×	×	×	
KS16-09	40	2016	11	19	24.00	136.99	66	94	27.77	×	×	×	×	×	×	×	
KS16-09	44	2016	11	18	24.23	140.25	55	128	26.32	×	×	×	×	×	×	×	
RF17-01	32	2017	1	12	23.74	133.00	29	94	24.99	×	×	×	×	×	×	×	
RF17-01	37	2017	1	11	24.25	135.01	46	105	25.47	×	×	×	×	×	×	×	

RF17-01	40	2017	1	11	23.98	137.01	98	112	23.30	×	×	×	×	×	×	×
RF17-01	44	2017	1	10	24.24	140.28	88	107	23.84	×	×	×	×	×	×	×
KS17-05	32	2017	6	20	23.76	133.00	14	112	27.35	×	×	×	×	×	×	×
KS17-05	37	2017	6	19	24.23	134.99	23	112	26.36	×		×	×	×	×	×
KS17-05	40	2017	6	19	23.99	137.00	21	102	27.92	×	×	×	×	×	×	×
KS17-05	44	2017	6	17	24.26	140.28	17	118	29.06	×	×	×	×	×	×	×
RF17-10	32	2017	12	9	23.76	133.00	101		27.02	×	×	×	×	×	×	×
RF17-10	37	2017	12	8	24.21	135.04	55	135	25.45	×	×	×	×	×	×	×
RF17-10	40	2017	12	8	23.99	137.00	77	92	25.14	×	×	×	×	×	×	×
RF17-10	44	2017	12	6	24.24	140.27	88	118	26.62	×	×	×	×	×	×	×
KS18-05	32	2018	6	1	23.74	133.00	12	102	27.50	×		×	×	×	×	
KS18-05	37	2018	5	31	24.25	135.01	11	105	29.40	×		×	×	×	×	
KS18-05	40	2018	5	30	23.98	136.93	16	107	28.71	×		×	×	×	×	
KS18-05	44	2018	5	29	24.25	140.26	19	124	28.65	×		×	×	×	×	
KS19-04	32	2019	5	3	23.74	133.00	33	124	24.37	×	×	×	×	×	×	
KS19-04	37	2019	5	2	24.24	135.00	15	92	24.73	×	×	×	×	×	×	
KS19-04	40	2019	5	1	23.96	137.00	22	125	27.19	×	×	×	×	×	×	
KS19-04	44	2019	4	30	24.24	140.26	19	98	23.50	×	×	×	×	×	×	

×: the observation was conducted.

*KS and RF in the cruise names indicate R/V Keifu-maru and R/V Ryofu-maru, respectively.

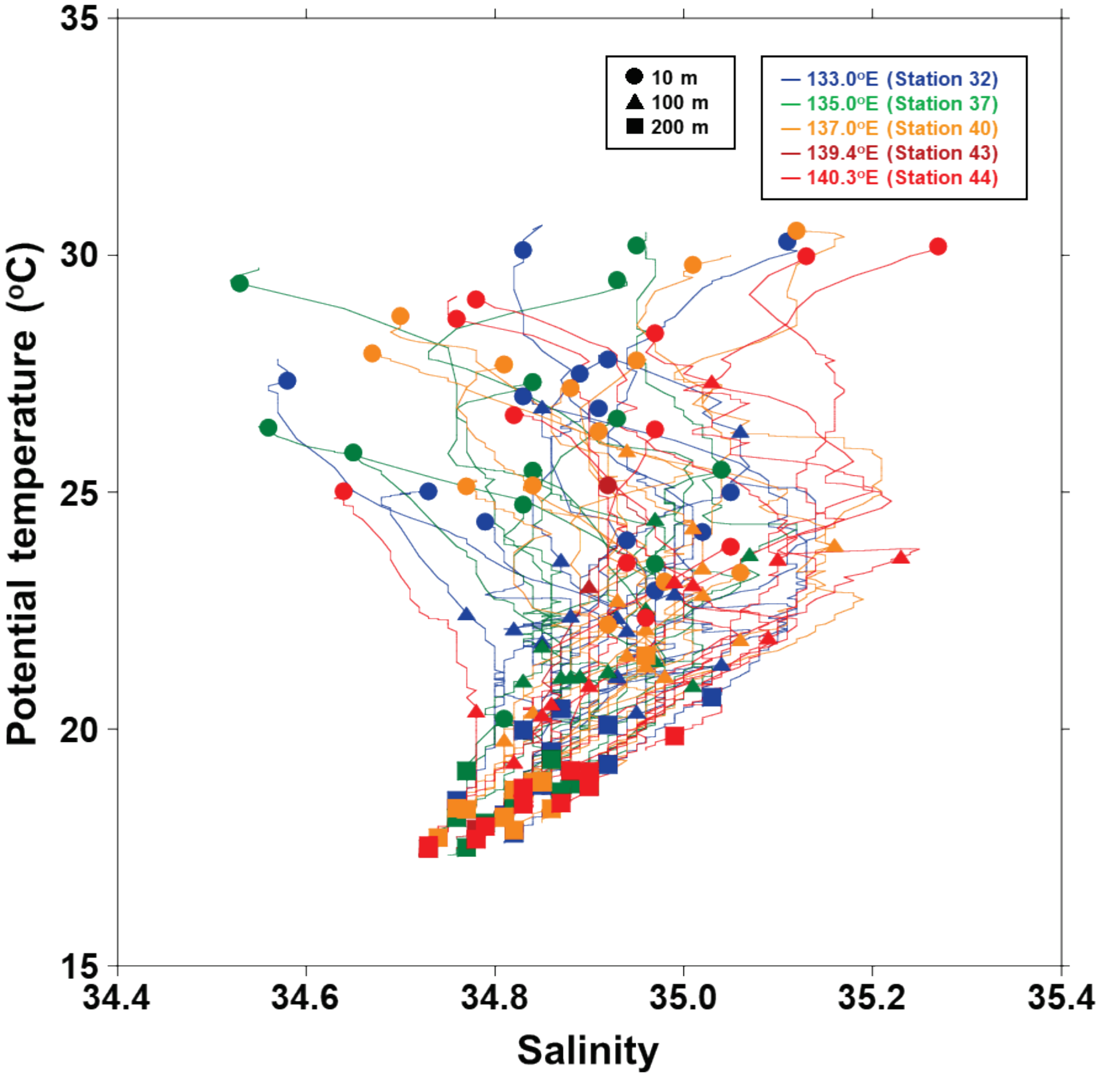

Supplementary Fig. 1. Temperature-salinity (T-S) diagram for all sampling stations. Data on the 1-m interval of potential temperature and salinity are depicted as the colored lines. The color indicates the longitude (°E) of sampling stations. The data on 10-, 100-, and 200-m depths are marked by the solid symbols with circles, triangles, and squares, respectively. Potential temperature and salinity at 10-, 100- and 200-m depths were not significantly different among the stations (Friedman test, $p>0.05$).

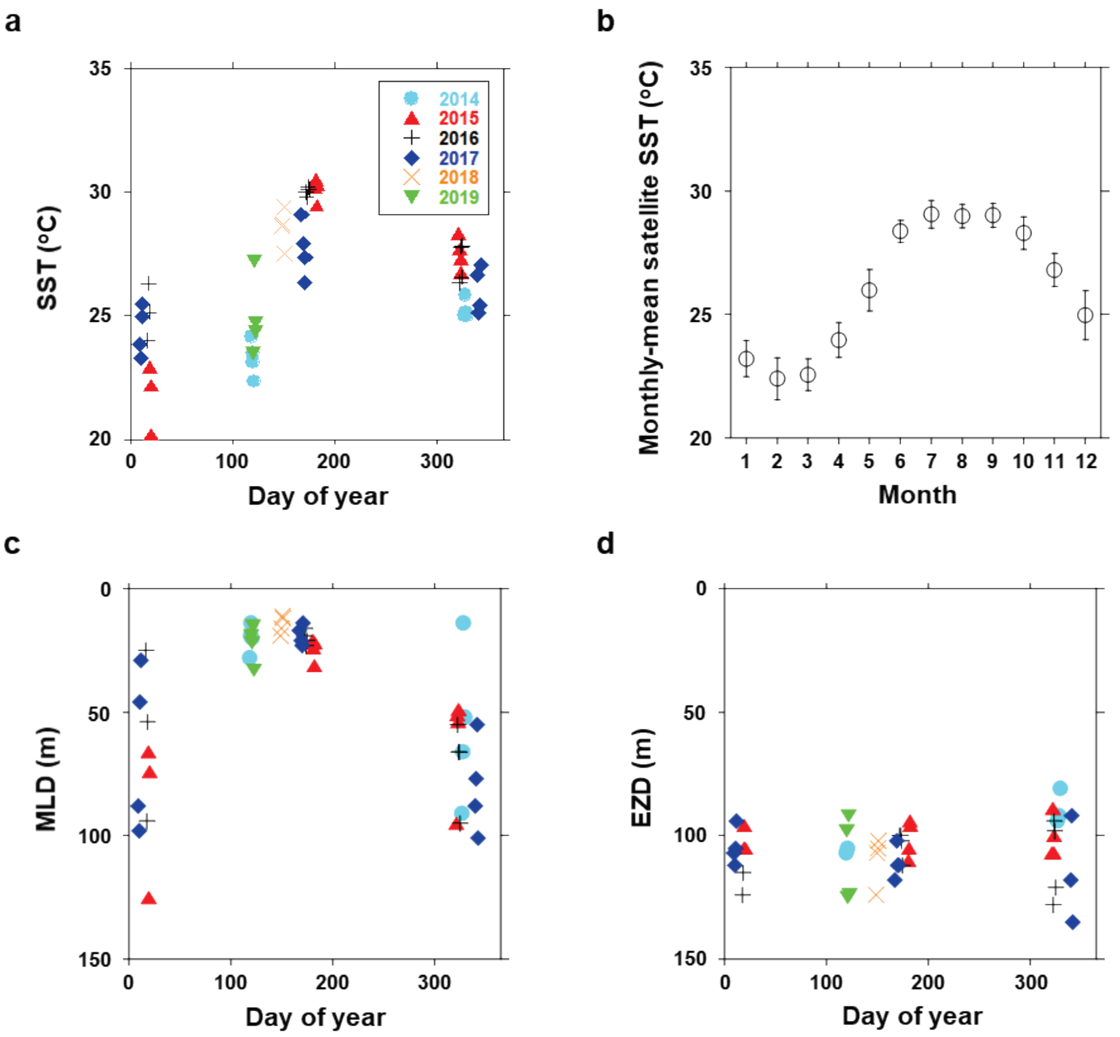

Supplementary Fig. 2. Seasonal trends of sea surface temperature (SST), mixed layer depth (MLD), and euphotic zone depth (EZD) in the study region. a, **c**, **d**, SST (**a**), MLD (**c**), and EZD (**d**) obtained from time-series *in situ* observation. The colored symbols indicate the observed years. **b**, Monthly-mean satellite SST during the period from April 2014 to May 2019. The error bars denote the 95% confidence interval (CI) (*n*=6 for April and May; *n*=5 for other months).

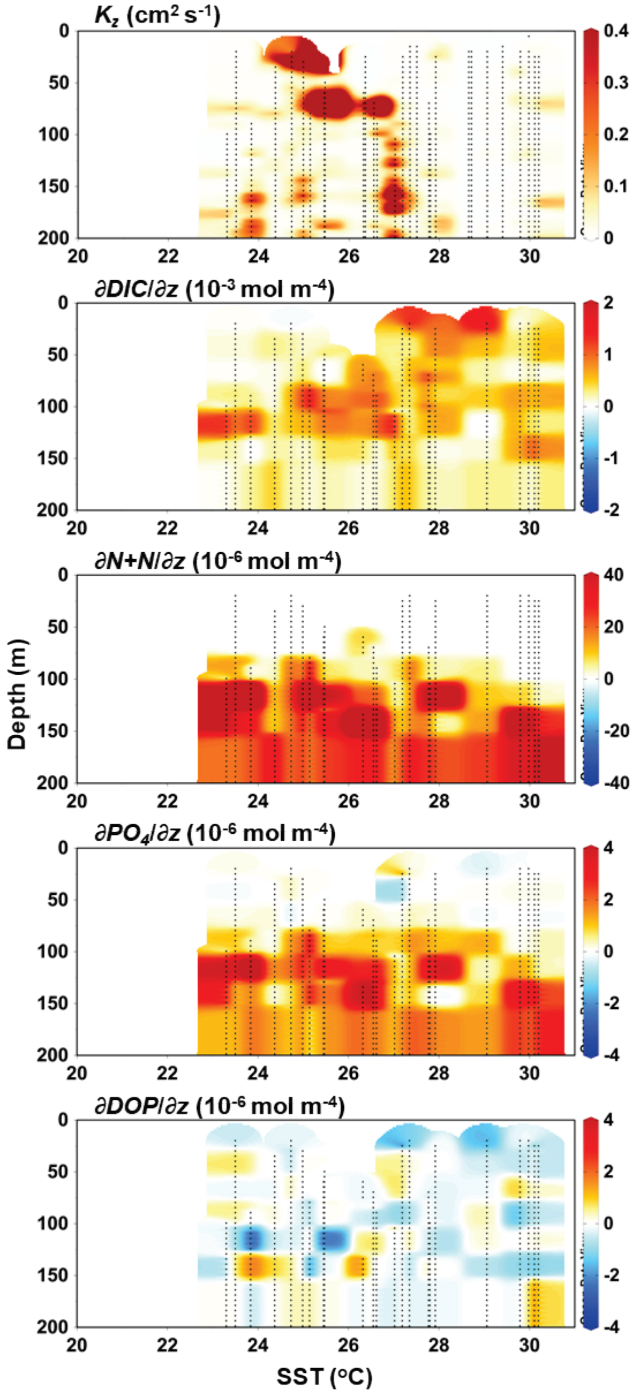

Supplementary Fig. 3. Vertical profiles of K_z, ∂*DIC*/∂*z*, ∂*N+N*/∂*z*, ∂PO_4/∂*z*, and ∂*DOP*/∂*z* in the layer between the mixed layer depth (MLD) and 200-m depth plotted against sea surface temperature (SST). The small black dots denote the depths where K_z data were obtained. The color contours were drawn by the weighted-average griding of Ocean Data View (ver. 5.2.1) with a x-y scale-length of 40-30.

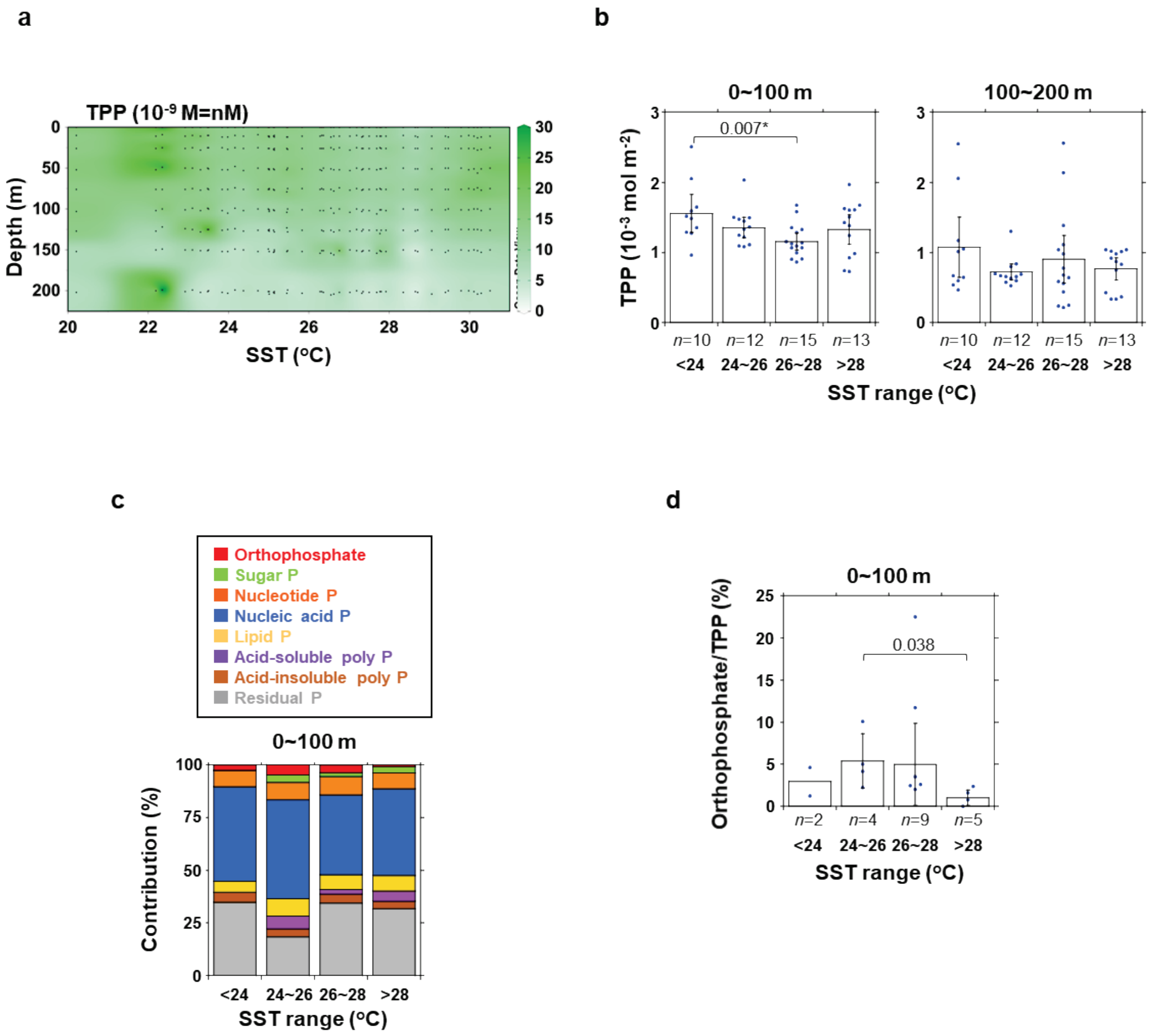

Supplementary Fig. 4. Vertical distributions of total particulate phosphorus (TPP) and its composition over sea surface temperature (SST). a, Vertical distribution of TPP concentrations in the upper 200 m of the water column plotted against SST. The small black dots denote the sampling depths. The color contours were drawn by the weighted-average griding of Ocean Data View (ver. 5.2.1) with a x-y scale-length of 65-65. **b**, Integrated stocks of TPP within the 0~100 m (left) and 100~200 m (right) layers in four SST regimes. Bar charts represent mean values of the integrated stocks indicated as blue dots. The error bars denote the 95% confidence interval (CI). Significant differences (two-sided Kruskal-Wallis test with Dunn's multiple comparisons, p<0.0083) in mean stocks between the SST regimes are depicted with an asterisk (*) just above the bars. **c**, The mean contributions of each P fraction to TPP within the 0~100 m layer in the four SST regimes. **d**, The contributions of orthophosphate to TPP

within the 0~100 m layer in the four SST regimes. Bar charts represent mean values of the orthophosphate percentages indicated as blue dots. The error bars denote the 95% CI. The mean percentage of the SST<24°C regime was obtained from two stations (no 95% CI). Significant differences (two-sided Kruskal-Wallis test, $p<0.05$) in the mean percentages between three SST regimes (except for the SST<24°C regime) are stated just above the bars.

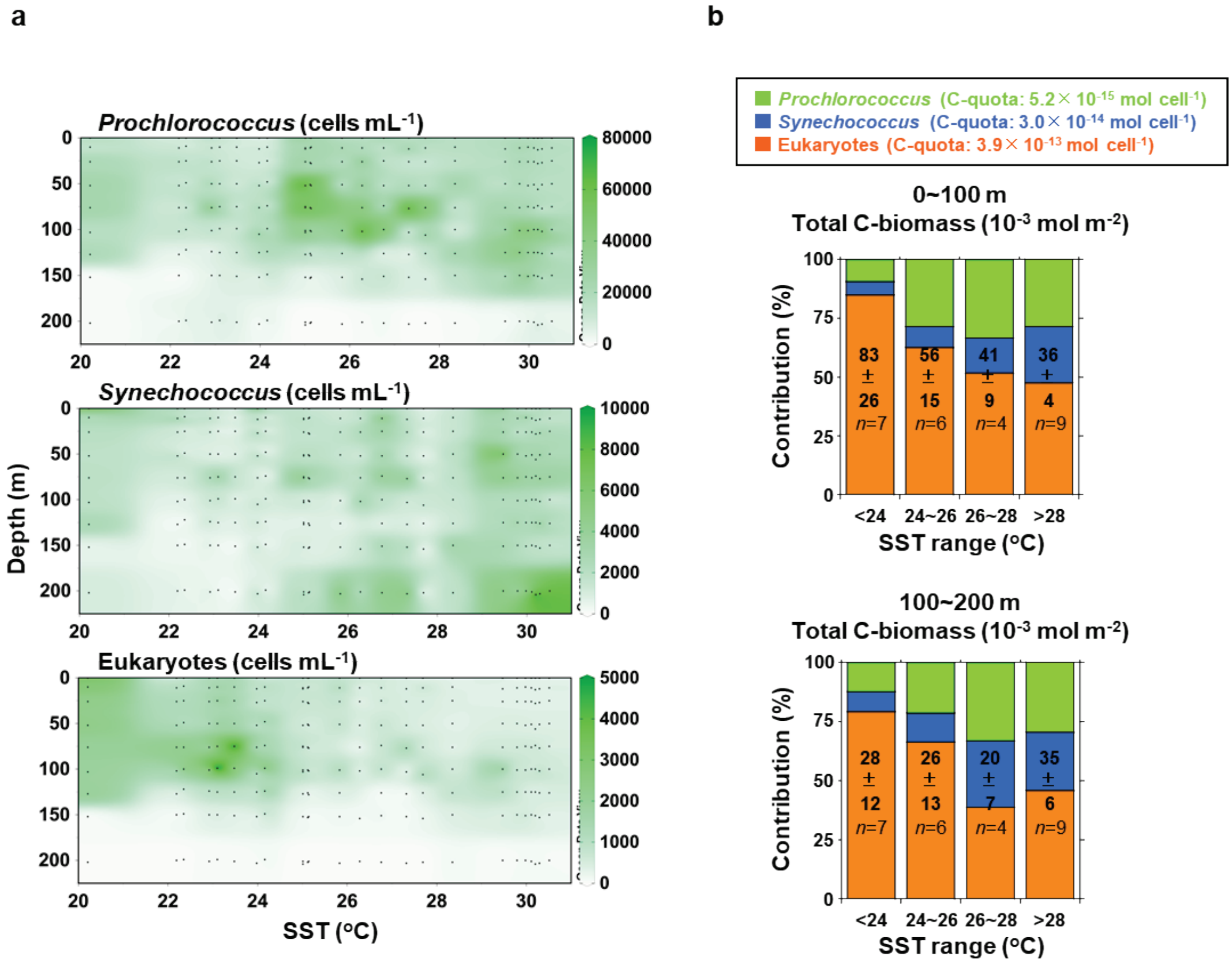

Supplementary Fig. 5. Distributions of microbes over sea surface temperature (SST). a, Vertical distributions of *Prochlorococcus*, *Synechococcus*, and pico- and nano-sized eukaryotes in the upper 200 m of the water column plotted against SST. The small black dots denote the sampling depths. The color contours were drawn by the weighted-average griding of Ocean Data View (ver. 5.2.1) with a x-y scale-length of 65-65. **b**, The mean contributions of each group to total C biomass of these groups within the 0~100 m (top) and the 100~200 m (bottom) layers in four SST regimes. The C biomass of each group was calculated using their cellular C quotas as denoted with the legends (Methods). The total C biomass of these groups in each SST regime (mean±95% confidence interval) is indicated in the panels.

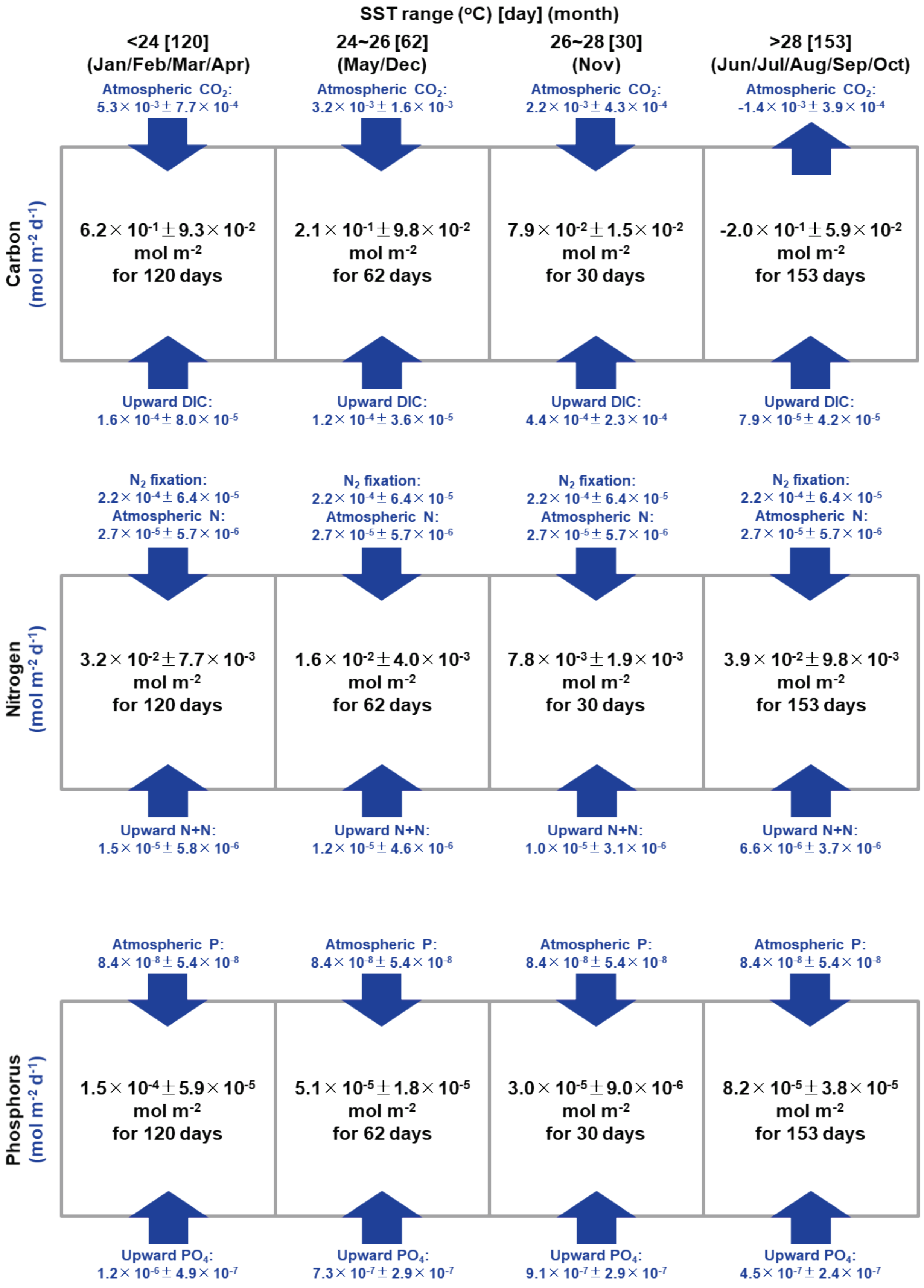
SST range (°C) [day] (month)
<24 [120] (Jan/Feb/Mar/Apr)
24~26 [62] (May/Dec)
26~28 [30] (Nov)
>28 [153] (Jun/Jul/Aug/Sep/Oct)
Carbon (mol m^{-2} d^{-1})
Atmospheric CO_2: $5.3\times10^{-3}\pm7.7\times10^{-4}$
Atmospheric CO_2: $3.2\times10^{-3}\pm1.6\times10^{-3}$
Atmospheric CO_2: $2.2\times10^{-3}\pm4.3\times10^{-4}$
Atmospheric CO_2: $-1.4\times10^{-3}\pm3.9\times10^{-4}$
$6.2\times10^{-1}\pm9.3\times10^{-2}$ mol m^{-2} for 120 days
$2.1\times10^{-1}\pm9.8\times10^{-2}$ mol m^{-2} for 62 days
$7.9\times10^{-2}\pm1.5\times10^{-2}$ mol m^{-2} for 30 days
$-2.0\times10^{-1}\pm5.9\times10^{-2}$ mol m^{-2} for 153 days
Upward DIC: $1.6\times10^{-4}\pm8.0\times10^{-5}$
Upward DIC: $1.2\times10^{-4}\pm3.6\times10^{-5}$
Upward DIC: $4.4\times10^{-4}\pm2.3\times10^{-4}$
Upward DIC: $7.9\times10^{-5}\pm4.2\times10^{-5}$
Nitrogen (mol m^{-2} d^{-1})
N_2 fixation: $2.2\times10^{-4}\pm6.4\times10^{-5}$ Atmospheric N: $2.7\times10^{-5}\pm5.7\times10^{-6}$
N_2 fixation: $2.2\times10^{-4}\pm6.4\times10^{-5}$ Atmospheric N: $2.7\times10^{-5}\pm5.7\times10^{-6}$
N_2 fixation: $2.2\times10^{-4}\pm6.4\times10^{-5}$ Atmospheric N: $2.7\times10^{-5}\pm5.7\times10^{-6}$
N_2 fixation: $2.2\times10^{-4}\pm6.4\times10^{-5}$ Atmospheric N: $2.7\times10^{-5}\pm5.7\times10^{-6}$
$3.2\times10^{-2}\pm7.7\times10^{-3}$ mol m^{-2} for 120 days
$1.6\times10^{-2}\pm4.0\times10^{-3}$ mol m^{-2} for 62 days
$7.8\times10^{-3}\pm1.9\times10^{-3}$ mol m^{-2} for 30 days
$3.9\times10^{-2}\pm9.8\times10^{-3}$ mol m^{-2} for 153 days
Upward N+N: $1.5\times10^{-5}\pm5.8\times10^{-6}$
Upward N+N: $1.2\times10^{-5}\pm4.6\times10^{-6}$
Upward N+N: $1.0\times10^{-5}\pm3.1\times10^{-6}$
Upward N+N: $6.6\times10^{-6}\pm3.7\times10^{-6}$
Phosphorus (mol m^{-2} d^{-1})
Atmospheric P: $8.4\times10^{-8}\pm5.4\times10^{-8}$
Atmospheric P: $8.4\times10^{-8}\pm5.4\times10^{-8}$
Atmospheric P: $8.4\times10^{-8}\pm5.4\times10^{-8}$
Atmospheric P: $8.4\times10^{-8}\pm5.4\times10^{-8}$
$1.5\times10^{-4}\pm5.9\times10^{-5}$ mol m^{-2} for 120 days
$5.1\times10^{-5}\pm1.8\times10^{-5}$ mol m^{-2} for 62 days
$3.0\times10^{-5}\pm9.0\times10^{-6}$ mol m^{-2} for 30 days
$8.2\times10^{-5}\pm3.8\times10^{-5}$ mol m^{-2} for 153 days
Upward PO_4: $1.2\times10^{-6}\pm4.9\times10^{-7}$
Upward PO_4: $7.3\times10^{-7}\pm2.9\times10^{-7}$
Upward PO_4: $9.1\times10^{-7}\pm2.9\times10^{-7}$
Upward PO_4: $4.5\times10^{-7}\pm2.4\times10^{-7}$

Supplementary Fig. 6. The C, N, and P fluxes in four different sea surface temperature (SST) regimes. Each box represents the water column (0~100 m depth). The blue arrows with blue text indicate the influxes or effluxes. The numbers of days and related months for each SST regime are indicated with the SST ranges. Total net fluxes in each SST regime are stated with black text within the boxes. The errors of the stated values denote the 95% confidence interval (CI). The procedures for calculating the stated values are described in the main text and Methods.

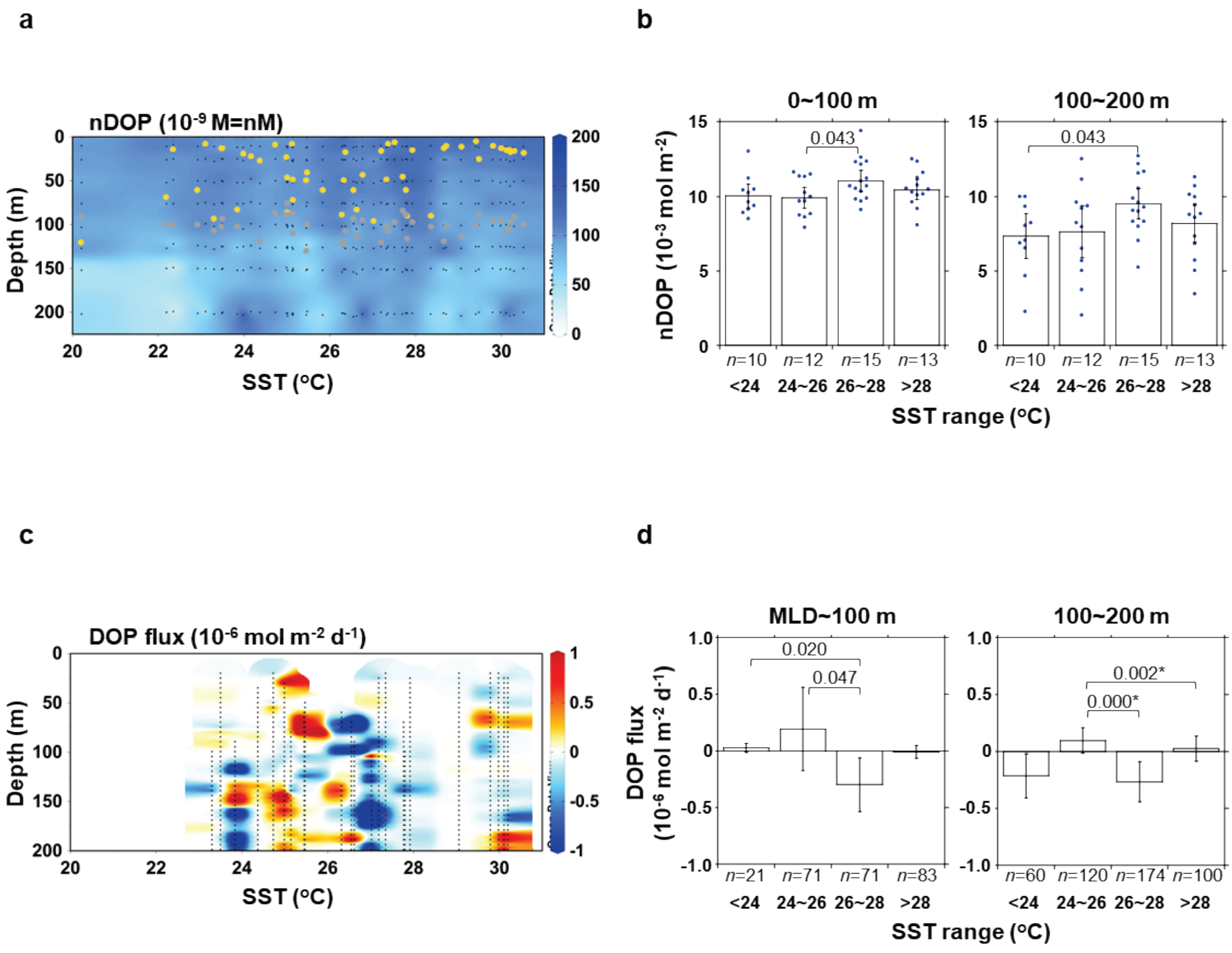

Supplementary Fig. 7. Distribution of the salinity (34.91) normalized dissolved organic phosphorus (nDOP) and the vertical flux of DOP in the upper 200 m over sea surface temperature (SST). a, Vertical distributions of nDOP concentrations plotted against SST. The small black dots denote the sampling depths. The yellow and gray solid circles indicate the mixed layer depth (MLD) and euphotic zone depth (EZD), respectively. The color contours were drawn by the weighted-average griding of Ocean Data View (ver. 5.2.1) with a x-y scale-length of 65-65. **b**, Integrated stocks of nDOP in four SST regimes within the 0~100 m (left) and 100~200 m (right) layers. Bar charts represent mean values of integrated stocks indicated as blue dots. The error bars denote the 95% confidence interval (CI). Significant differences (two-sided Kruskal-Wallis test, $p<0.05$) in the mean stocks between the SST regimes are depicted just above the bars. **c**, Vertical profiles of DOP fluxes in the layer between the MLD and 200-m depth plotted against SST. The small black dots denote the depths where K_z data were obtained. The color contours were drawn by the weighted-average griding of Ocean Data View (ver. 5.2.1) with a x-y scale-length of 40-30. **d**, Mean fluxes of DOP in the

four SST regimes within the MLD~100 m (left) and 100~200 m (right) layers shown in **c**. The error bars denote the 95% CI. Significant differences (two-sided Kruskal-Wallis test, $p<0.05$) in the mean fluxes between the SST regimes are stated just above the bars. Significant differences of multiple comparisons with the Dunn's procedure after the Kruskal-Wallis test ($p<0.0083$) are marked with an asterisk (*).

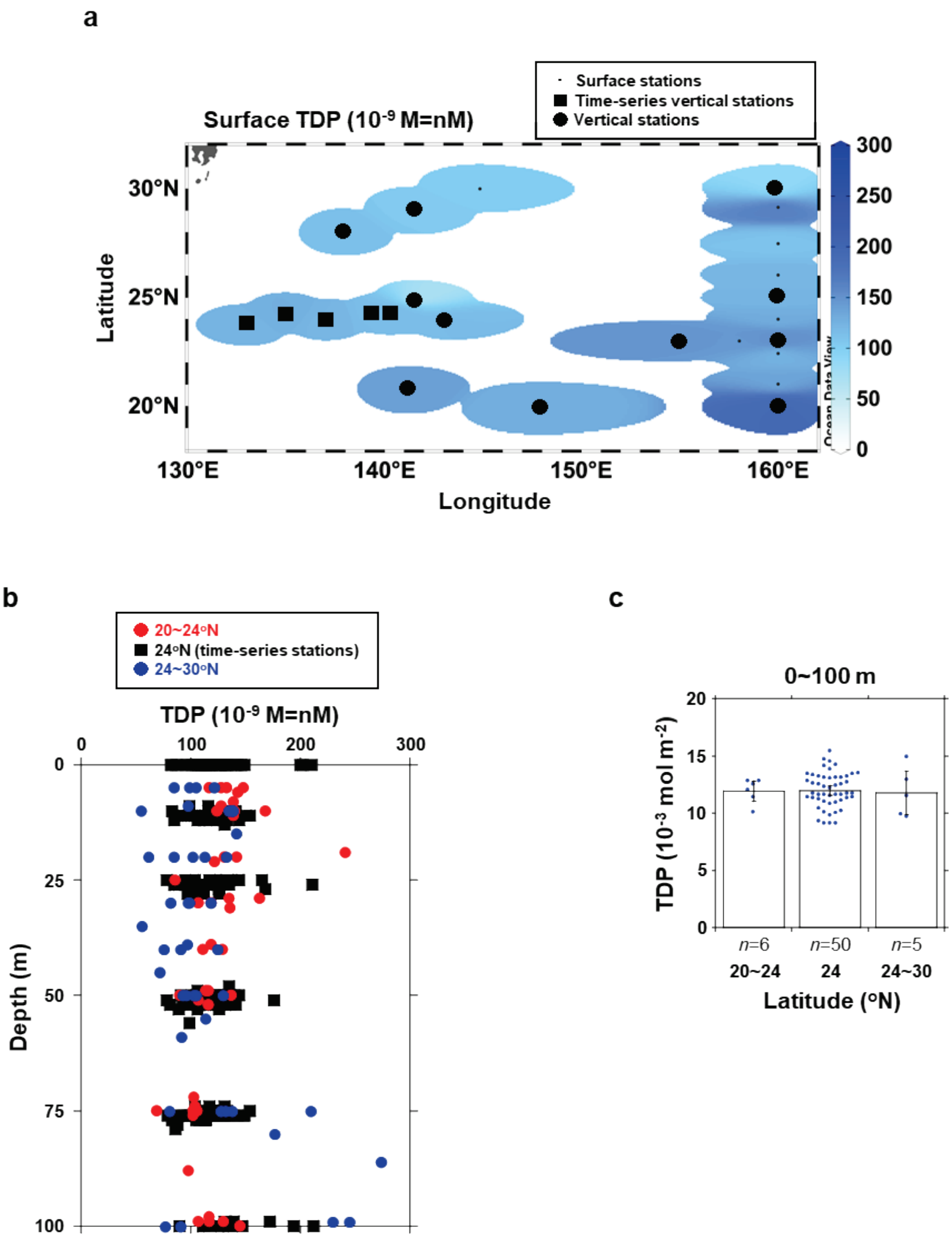

Supplementary Fig. 8. Horizontal distributions of total dissolved phosphorus (TDP: DOP+PO_4) in the western subtropical North Pacific. a, Surface distribution (<10 m) of TDP concentrations compiled from our previous studies and this study. At the stations with the small black dots, only surface sampling was conducted. The black

squares and black circles denote vertical sampling stations of this study (on a 24°N transect) and our previous studies, respectively. The color contours were drawn by the weighted-average griding of Ocean Data View (ver. 5.2.1) with a x-y scale-length of 45-50. **b**, Vertical profiles of TDP concentrations in the 0~100 m layer at the stations of 20~24°N (red circles), 24°N transect (black squares), and 24~30°N (blue circles). **c**, Integrated stocks of TDP in the 0~100 m layer at the stations of 20~24°N, 24°N transect, and 24~30°N. Bar charts represent mean values of the integrated stocks indicated as blue dots. The error bars denote the 95% confidence interval (CI). The mean stocks between the stations were not significantly different (two-sided Kruskal-Wallis test, p>0.05).
